# The Determining Role of Nb Interlayer on Interfacial Microstructure and Mechanical Properties of Ti/Steel Clad Plate by Vacuum Rolling Cladding

**DOI:** 10.3390/ma11101983

**Published:** 2018-10-15

**Authors:** Guang-ming Xie, De-han Yang, Zong-an Luo, Ming Li, Ming-kun Wang, R. D. K. Misra

**Affiliations:** 1 State Key Laboratory of Rolling and Automation, Northeastern University, Shenyang 110819, Liaoning, China; luoza@ral.neu.edu.cn (Z.-a.L.); 13840548253@163.com (M.L.); 13504769957@163.com (M.-k.W.); 2 Department of Metallurgical, Materials and Biomedical Engineering, University of Texas at El Paso, 500 W. University Avenue, El Paso, TX 79968 USA; dmisra2@utep.edu

**Keywords:** vacuum rolling cladding (VRC), Ti/steel clad plate, shear property, Nb interlayer, interfacial compound

## Abstract

We elucidate here the determining role of Nb interlayer on mechanical properties of Ti/steel clad plate fabricated by vacuum rolling cladding (VRC) as a function of different heating temperatures. A critical analysis on the clad interface via electron probe micro-analyzer, X-ray diffractometer and shear testing were conducted to investigate the influence of TiC, Fe-Nb and TiFe compounds and Nb-Ti solid solution on microstructural evolution and shear properties of Ti/steel clad plate. The inter-diffusion between Ti, C and Fe was effectively restrained by adding the Nb interlayer at heating temperature of 800 °C, and average shear strength of 279 MPa was achieved. With increase of heating temperature, Nb-Ti solid solution was formed at the Ti/Nb interface, which reduced mechanical properties of clad plate at 900 °C. At 1000 °C, TiC and Nb-Fe compounds and Nb-Ti solid solution were formed at the interface, and minimum average shear strength of 152 MPa was achieved. The detailed analysis on the clad interface suggested that ideal shear strength can be obtained through the addition of Nb interlayer and selecting appropriate heating temperature.

## 1. Introduction

As a metal with high corrosion resistance, titanium is widely used in some fields including petrochemical, ship building, marine engineering, electric power industry etc. However, the application of titanium and titanium alloys is remarkably limited because of relatively high price. The Ti/steel clad plate addresses this aspect. It not only ensures the perfect corrosion-resistance of Ti, but also provides excellent mechanical properties of ordinary steel plate, improves the service life of the equipment, and greatly reduces the production cost [1,2,3,4,5,6]. A few studies in this regard have been carried out on the manufacturing Ti/steel clad plate [7,8,9].

Currently, there are four primary ways of fabricating Ti/steel clad plate, which include explosive cladding, diffusion cladding, conventional hot-rolling cladding and vacuum rolling cladding (VRC). The four preparation methods have their own advantages and disadvantages. The explosive cladding used to produce Ti/steel clad plate is rather convenient, but its application is significantly restricted by low and non-uniform interface bonding of the clad plate and environmental pollution [10,11,12]. Compared with the explosive cladding method, the diffusion cladding is environmentally friendly, but it exhibits long diffusion time, lower production efficiency and lower bonding strength [13,14]. Recently, the VRC technology was developed by introducing vacuum electron beam welding technology (EBW) on the basis of traditional hot-rolling cladding method. Brittle interfacial products such as various oxides and nitrides can be effectively inhibited by VRC, which are expected to improve the mechanical properties of the clad plate. Furthermore, the VRC process is simple, and wider and thicker clad plate products could be produced easily. Therefore, VRC technique is being mostly adopted [15,16,17,18].

During hot-rolling cladding Ti/steel clad plate, the oxides and nitrides at the interface can be effectively controlled by VRC, but TiFe and TiC compounds are easily produced at the interface of Ti and steel at elevated temperature, which reduce the interfacial bonding property in Ti/steel clad plate. In order to reduce the contents of TiC and TiFe and increase interfacial bonding strength, intermediate layer metals are commonly applied [19]. Recent some studies focused on mechanical properties of Ti/steel clad interface with various interlayers. Eroglu et al. [20] studied diffusion bonding between Ti-6Al-4V alloy and micro-duplex stainless steel with copper interlayer, and the results indicated that TiFe cannot be eliminated by copper interlayer. Kundu and Chatterjee [21] analyzed the effect of temperature on microstructurial evolution and mechanical properties of Ti/Nb/stainless steel interface via diffusion cladding. The results noted the presence of Fe_2_Nb and Fe_7_Nb_6_ under different temperature conditions. Yu et al. [22] studied the shear properties and microstructure of TA2/Q235B interface through hot-rolling cladding with DT4-alloy layer, demonstrating the formation of TiFe intermetallic compound at the interface at low temperature. In this study, the effect of Nb interlayer on microstructural evolution and shear strength of Ti/steel clad plate by VRC is explored at various heating temperatures.

## 2. Experimental

### 2.1. Preparation of Raw Materials

The chemical composition of commercial purity titanium (TA2) plate and Q345 substrate steel plate are given in Table 1. The 80 μm thick, 99.8 wt.% Nb foil was as the metal interlayer. The dimensions of Q345 plates were 150 mm × 100 mm × 20 mm and 150 mm × 100 mm × 40 mm, while the dimensions of titanium plates and Nb foils were 126 mm × 76 mm × 10 mm and 126 mm × 76 mm × 0.08 mm, respectively. The original surfaces of steel and Ti plates were treated by traditional grinding and buffing techniques to exposing clear metal surface. Meanwhile, Nb foil of 80 μm in thickness was ground using sand paper and cleaned in an ultrasonic bath containing acetone after brushing.

### 2.2. Vacuum Electron Beam Welding Process

**Figure 1:**
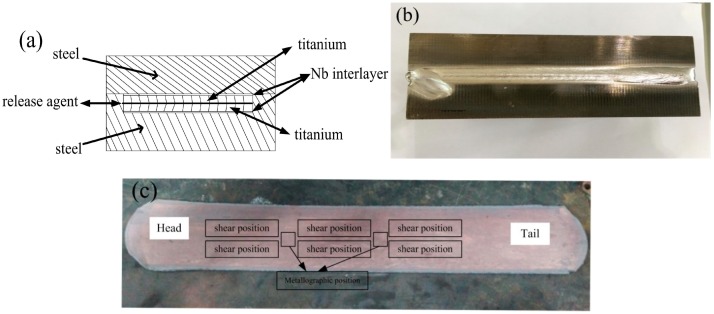
Principle drawing of the assembling Ti/steel (**a**); blank morphology after welding (**b**) and samples locations for microstructure and mechanical testing (**c**).

To making the high interfacial vacuum level, the assembly was carefully designed as shown in Figure 1a, and morphology of the sample after welding is displayed in Figure 1b. Metallographic and mechanical test samples are exhibited in Figure 1c. A20 mm groove in depth was obtained in the center of 40 mm thick steel plate. A release agent, which was composed of Na_2_SiO_3_·9H_2_O powder solution, was used to separate the two layers of Ti plates intimately bonded during symmetrical rolling. Meanwhile, Nb foil was placed between the titanium plate and the groove. The other Nb foil was placed between titanium plate and the other steel plate of 20 mm in thickness. To ensure high interfacial vacuum level, the two steel substrate plates were electron beam joined within a vacuum chamber before cladding. During EBW a beam current of 65 mA, high voltage of 85 kV and traverse speed of 280 mm/min in vacuum level of1 × 10^−2^ Pa were adopted.

### 2.3. Hot Roll Bonding

The titanium/steel blank by EBW was subsequently heated in a furnace in the range of 800–1000 °C for 2 h. The Ti/steel blank was hot-rolledvia7deformation passes, and the total reduction reached 85%, which was helpful to obtain excellent metallurgical bonding due to severe rolling deformation. The detailed rolling and temperature parameters were illustrated in Table 2.

### 2.4. Evaluation of Interfacial Structure and Mechanical Properties

Sampling direction for microstructural examination and tensile shear testing is corresponded to the hot rolling direction (Figure 1c). The microstructural specimens with the clad interface was ground with sand paper up to 1500 grit and polished with SiO_2_ suspension followed by etching with a mix solution of 5 mL nitric acid and 95 mL ethanol. The microstructures of the clad interface and shear fractural surface was characterized by field emission electron probe micro-analyzer (EPMA, 8530F, JEOL, Tokyo, Japan) equipped with wavelength dispersive X-ray spectrometer (WDS). The constituents of interfacial compound on the fractural surface were analyzed using X-ray diffractometer (XRD, D-Max2500, JEOL, Tokyo, Japan).

The shear strength test of Ti/steel clad plate is according to the ASTM B898 specification [23]. Six shear specimens for every kind of Ti/steel clad plates were prepared and tested to achieve the average shear strength. The tensile strain rate was maintained at 1 × 10^−3^ s^−1^.

## 3. Results and Discussion

### 3.1. Microstructural Characterization of Bonded Interfaces

**Figure 2:**
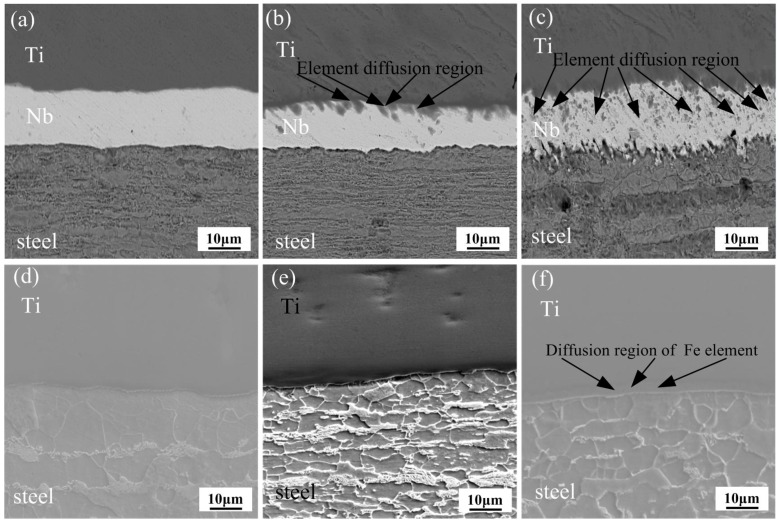
The backscattered electron (BSE) images of the interfaces at different heating temperatures: (**a**) 800 °C; (**b**) 900 °C and (**c**) 1000 °C for Ti/Nb/steel clad plate; (**d**) 800 °C; (**e**) 900 °C and (**f**) 1000 °C for Ti/steel clad plate.

**Figure 3:**
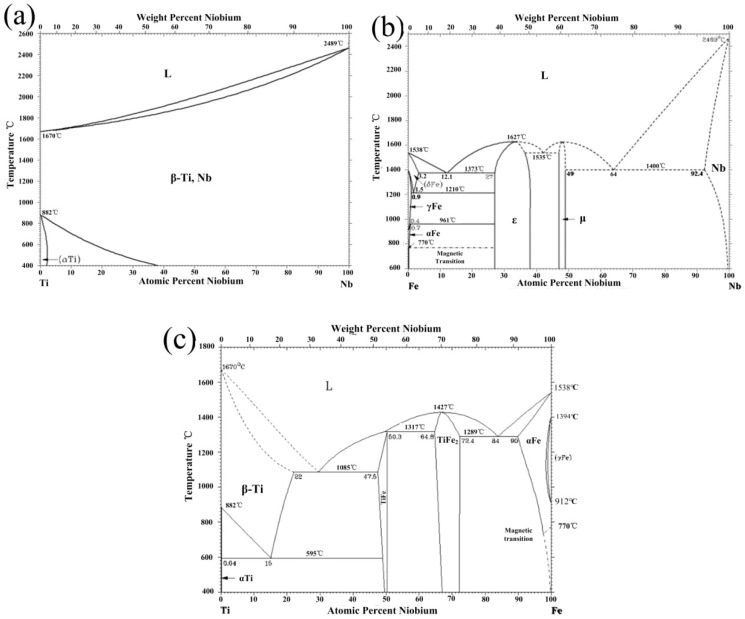
The binary phase diagrams of different elements: (**a**) Ti-Nb; (**b**) Fe-Nb and (**c**) Ti-Fe.

The backscattered electron (BSE) images of the clad interface for Ti/Nb/steel at different heating temperatures are shown in Figure 2a–c. The apparent element diffusion area was not found at the Nb/steel and Ti/Nb interfaces at 800 °C in Figure 2a. With increase of heating temperature, the non-uniform diffusion area of elements was formed at the Ti/Nb interface at 900 °C in Figure 2b, which was mainly consisted of Ti (38.2–40.9 wt.%) and Nb (58.1–60.2 wt.%) based on WDS result. Thus, Nb-Ti solid solution may be produced at the Ti/Nb interface by means of the binary phase diagram of Ti and Nb in Figure 3a. However, the apparent diffusion area of the element was not found at the Nb/steel interface at 900 °C. When the heating temperature was further increased, the inhomogeneous element diffusion area was obvious at the Ti/Nb and Nb/steel interfaces at 1000 °C in Figure 2c. According to WDS result, the Ti/Nb interface was mainly enriched with Ti (47.5–48.1 wt.%) and Nb (51.3–52.1 wt.%), and the Nb/steel interface was enriched with Nb (56.2–57.3 wt.%), Fe (32.8–33.3 wt.%) and Ti (10.6–11.2 wt.%). Nb-Fe intermetallic compound may be formed at the Nb/steel interface from the binary phase diagram of Fe and Nb in Figure 3b. Meanwhile, a number of elements diffused to niobium interlayer based on the BSE images in Figure 3c. Thus, Nb interlayer of 80 μm cannot effectively inhibit diffusion between Ti and Fe at 1000 °C.

The BSE images of the Ti/steel clad interface at various heating temperatures are revealed in Figure 2d–f. Micro-crack was not found at different heating temperature, and the interface was continuous. The apparent diffusion region of Fe was not discovered at 800 and 900 °C on the Ti side of interface in Figure 2d,e. However, the apparent diffusion region of Fe was found on the Ti side of interface at 1000 °C, which indicated that diffusion at the interface at 1000 °C in Figure 2f. Moreover, the diffusion region of Fe was enriched with Fe (15.7–16.1 wt.%) and Ti (83.2–84.1 wt.%). Therefore, the diffusion region of Fe element was β-Ti layer by means of the binary phase diagram of Ti and Fe in Figure 3c.

**Figure 4:**
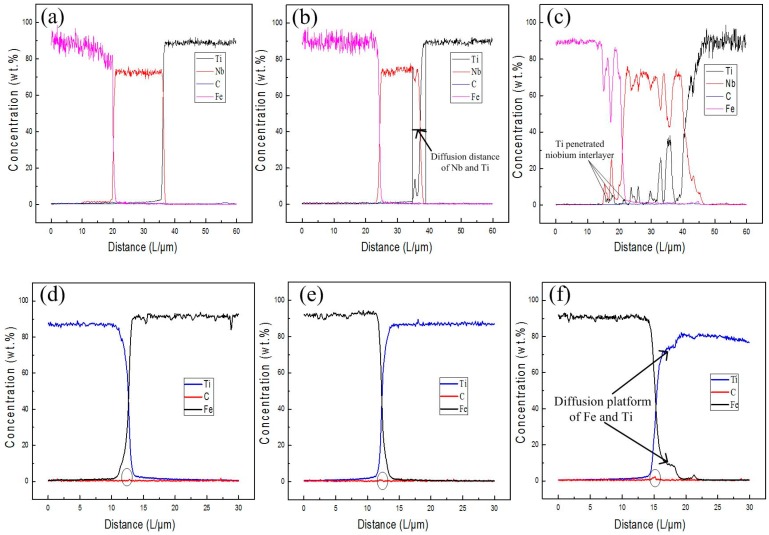
The WDS line scanning results traversing the clad interface at various heating temperatures: (**a**) 800 °C; (**b**) 900 °C and (**c**) 1000 °C for Ti/Nb/steel clad plate; (**d**) 800 °C; (**e**) 900 °C and (**f**) 1000 °C for Ti/steel clad plate.

The WDS line scanning results of Fe, C, Nb and Ti across the Ti/Nb and Nb/steel clad interface for different heating temperatures are represented in Figure 4a–c. In Figure 4a, the inter-diffusion of Ti and Fe elements was effectively restrained by the Nb interlayer, and there was no change in the content of carbon at the clad interface at 800 °C. With increase of heating temperature, 4 μm diffusion distance for both Ti and Nb elements was detected at the Ti/Nb interface. Previous studies have shown that α-β phase transformation point of pure Ti is 882 °C and α-Ti is transformed to β-Ti at 900 °C [24,25]. Meanwhile, more amount of β-Ti were changed into α-Ti during the cooling process after rolling. However, Nb is a strong β-Ti stabilizing element, which enables more β-Ti to be retained at the room temperature [21]. Thus, β-Ti was formed at the Ti/Nb interface in Figure 4b. The Nb interlayer cannot effectively restrain the inter-diffusion of Ti and Fe at 1000 °C in Figure 4c. At the Ti/Nb interface, the diffusion distance between Nb and Ti is extended. Meanwhile, diffusion distance of Ti was more far at the Ti/Nb interface, which diffused through Nb interlayer to the steel side at 1000 °C. Because carbon steel and Nb is FCC and BCC structure in the temperature range studied, respectively [16], smaller diameter of Fe atom and larger lattice constant of Nb led to diffusion of Fe atoms to the Nb side, which is greater than the diffusion distance of Nb atoms to the steel side at the Nb/steel interface. In addition, Nb-Fe intermetallic compound and TiC may be produced on the steel side close to the Nb/steel interface depending on the binary phase diagram of Ti, Fe and Nb.

The WDS line scanning results of Fe, C and Ti elements through the Ti/steel interface for various heating temperatures are displayed in Figure 4d–f. The accumulation of C atoms at the interface region was relatively low at 800 and 900 °C. However, the C content was enhanced at 1000 °C in Figure 4f. Moreover, the contents of Ti and Fe were also changed at the interface with increase of heating temperature. Diffusion platform of Ti and Fe at the Ti/steel interface occurred at 1000 °C, which corresponded with the result in Figure 2f.

**Figure 5:**
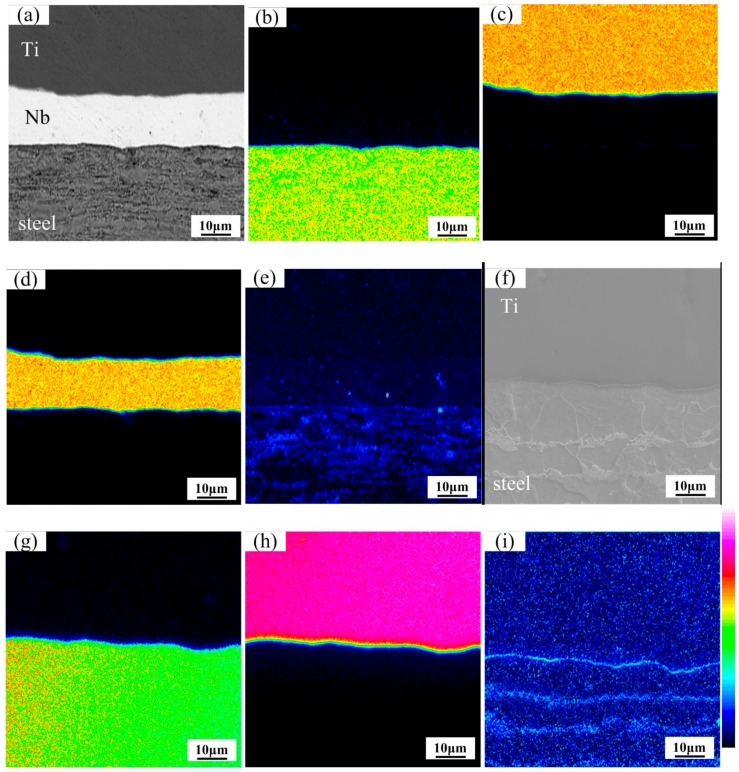
Map distribution micrographs of various elements at the interfaces at 800 °C: (**a**) BSE image; (**b**) Fe element; (**c**) Ti element; (**d**) Nb element and (**e**) C element for Ti/Nb/steel clad plate; (**f**) BSE image; (**g**) Fe element; (**h**) Ti element and (**i**) C element for Ti/steel clad plate.

The EPMA map analysis was used to study the diffusion of interfacial elements. The EPMA element map analysis at the Ti/steel interface with Nb interlayer is represented in Figure 5a–e at heating temperature of 800 °C. The apparent element diffusion was not found at the Ti/Nb and Nb/steel interfaces, which was similar to WDS line scanning and the BSE image results. Thus, Nb interlayer of 80 μm can effectively inhibit inter-diffusion between Ti, Fe and C at 800 °C.

The EPMA element map analysis at the Ti/steel interface without Nb interlayer is shown in Figure 5f–i. The diffusion of Ti and Fe was not quite obvious in Figure 5g,h. However, C was presented at the interface at 800 °C in Figure 5i. Thus, diffusion of Ti, C and Fe was effectively restrained by the Nb interlayer at 800 °C.

**Figure 6:**
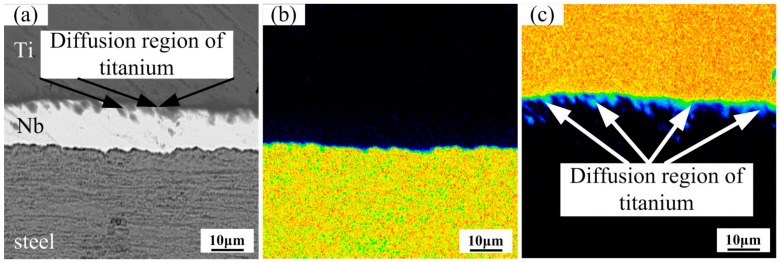

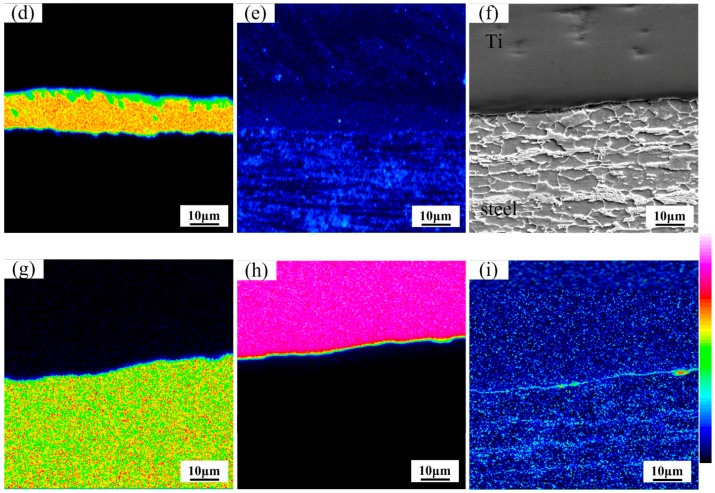
Map distribution micrographs of various elements at the interfaces at 900 °C: (**a**) BSE image; (**b**) Fe element; (**c**) Ti element; (**d**) Nb element and (**e**) C element for Ti/Nb/steel clad plate; (**f**) BSE image; (**g**) Fe element; (**h**) Ti element, and (**i**) C element for Ti/steel clad plate.

Figure 6a–e presents the element distribution of Ti/Nb/steel interface at heating temperature of 900 °C. The concentration gradient of both Nb and Ti was quite visible duo to the diffusion of Nb and Ti in this reaction layer. However, diffusion region of Fe or C elements was not detected at the Nb/steel interface in Figure 6b,e. In addition, Ti diffused into the Nb interlayer, but did not pass through the Nb interlayer in Figure 6c,d. Some studies [26,27] indicated that Ti-Fe intermetallic compounds and TiC are formed by the diffusion of elements at the interface of Ti/steel clad plate without interlayer, and the mechanical properties of the clad plate is seriously affected by these compounds. However, the Nb interlayer restricted the inter-diffusion between Ti, Fe and C and the formation of TiC and Ti-Fe compounds at heating temperature of 800 and 900 °C, which improved the mechanical properties of the clad plate in our study.

Figure 6f–i present the elemental distribution at Ti/steel interface at heating temperature of 900 °C. The diffusion of Ti and Fe was not obvious in Figure 6g,h, and the content of C started to increase at the interface in Figure 6i.

**Figure 7:**
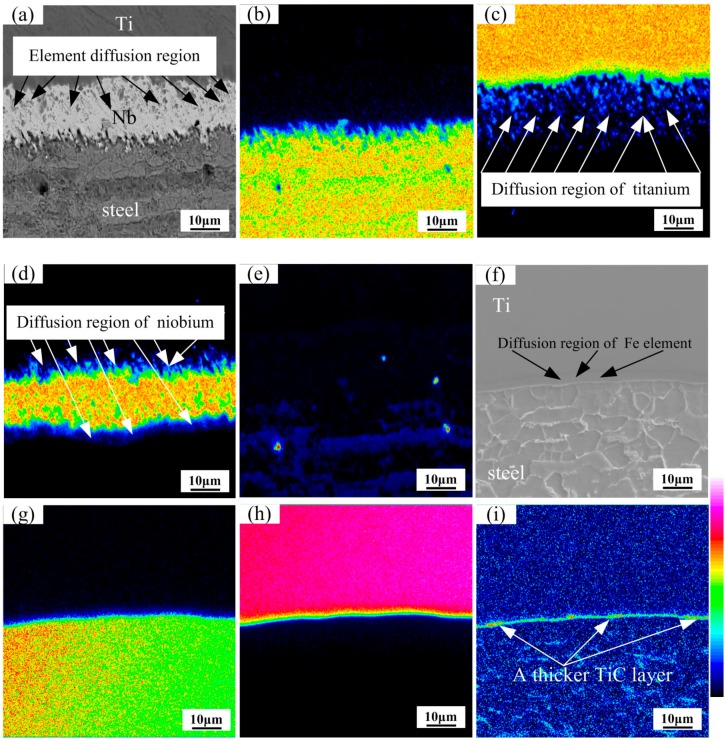
Map distribution micrographs of various elements at interface of the clad plates at 1000 °C. (**a**) BSE image; (**b**) Fe element; (**c**) Ti element; (**d**) Nb element and (**e**) C element for Ti/Nb/steel clad plate; (**f**) BSE image; (**g**) Fe element; (**h**) Ti element and (**i**) C element for Ti/steel clad plate.

The map distribution micrographs of various elements at the Ti/Nb/steel interface at heating temperature of 1000 °Care shown in Figure 7a–e. The diffusion concentration gradient of Nb, Fe and Ti in this reaction layer was clearly visible. Nb diffused to the titanium side and the steel side in Figure 7d. Meanwhile, diffusion region of Fe and C was not obvious at the Nb/steel interface in Figure 7b,e. However, the diffusion distance of Ti was large at the Ti/Nb interface, which diffused through the Nb interlayer until the steel side in Figure 7c. Nb interlayer cannot completely inhibit diffusion of Ti, and Ti diffuses through Nb interlayer at heating temperature of 1000 °C. Thus, TiC and Ti-Fe compounds may be produced near the Nb/steel interface, which reduced interfacial properties.

The map distribution micrographs of various elements at the Ti/steel interface at heating temperature of 1000 °C are shown in Figure 7f–i. The clear diffusion area in Fe and Ti elements was found near the Ti/steel interface in Figure 7f–h. Meanwhile, a thicker and consecutive TiC layer was formed in Figure 7i. Based on the above result, the element diffusion was sufficient at 1000 °C, and diffusion of C and Fe was effectively restrained by Nb interlayer at 1000 °C. However, diffusion of Ti was not restrained by Nb interlayer at 1000 °C, and Ti diffused through the Nb interlayer to the steel side.

### 3.2. Fractural Surface of the Bonded Interface

**Figure 8:**
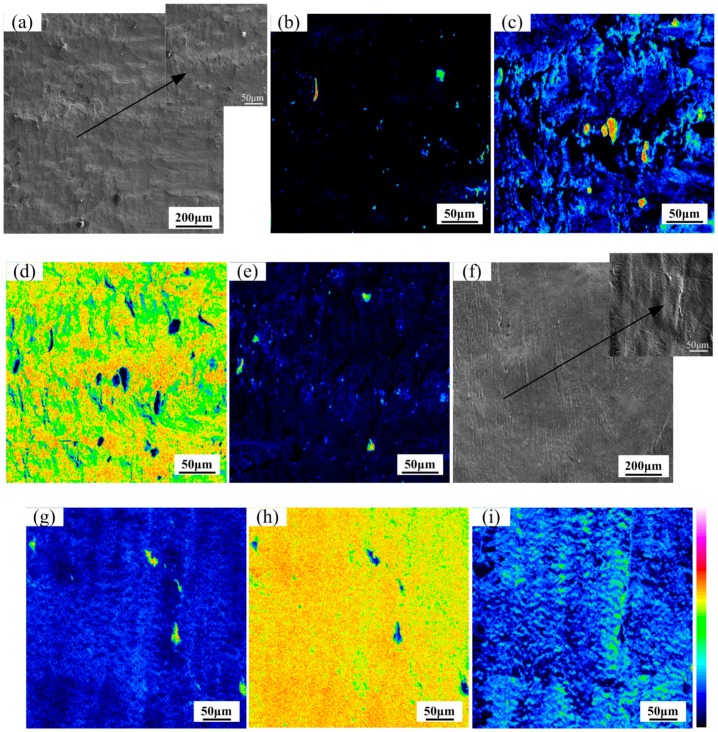
Map distribution micrographs of various elements on the fractural surface at 800 °C (Ti side): (**a**) BSE image; (**b**) Fe element; (**c**) Ti element; (**d**) Nb element and (**e**) C element for Ti/Nb/steel clad plate; (**f**) BSE image; (**g**) Fe element; (**h**) Ti element and (**i**) C element for Ti/steel clad plate.

The map distribution micrographs of various elements on the fractural surface of Ti/Nb/steel clad plate at heating temperature of 800 °C are presented in Figure 8a–e. The EPMA image shows the morphology of the fractural surface on the Ti side. The fractural surface (Figure 8a) had tearing marks with dimples, which suggested that the fractural mode at the interface was ductile at heating temperature of 800 °C. Furthermore, the fractural surface was almost covered by Nb based on the elemental distribution presented in Figure 8d. The EPMA micrographs of Fe, Ti and C distribution on the fractural surface are exhibited in Figure 8b,c and e. However, the contents of Fe and C were less than that of Ti, which indicated that the location of fracture was between Ti and Nb interlayer or at the Nb interlayer and was not at Nb/steel interface at heating temperature of 800 °C. In addition, the fractural surface was enriched with Nb (95.1–96.2 wt.%), Fe (0.1–0.8 wt.%), Ti (3.5–4.1 wt.%), C (0.5–1.2 wt.%) as indicated by WDS analysis, which also further explained that the position of fracture was at the Nb interlayer. Thus, Nb-Ti solid solution, TiC and Ti-Fe compound may be not formed at the clad interface with Nb interlayer at heating temperature of 800 °C, and the bonding was very good at the interface of Nb/Ti and Nb/steel.

**Figure 9:**
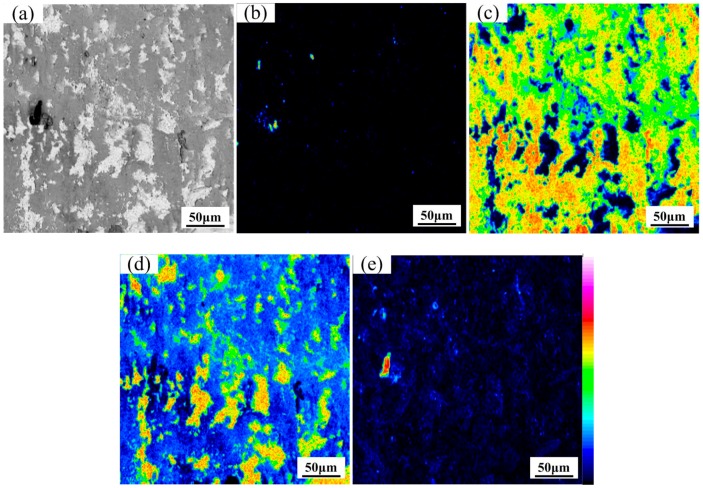
Map distribution micrographs of various elements on the fractural surface of Ti/Nb/steel clad plate at 900 °C (titanium side): (**a**) BSE image; (**b**) Fe element; (**c**) Ti element; (**d**) Nb element and (**e**) C element.

The map distribution micrographs of various elements on the fractural surface of Ti/steel clad plate at heating temperature of 800 °C are presented in Figure 8f–i. A number of tear bands in Figure 9f suggested that the fractural mode at the Ti/steel interface was ductile at heating temperature of 800 °C. In addition, the fractural surface was almost filled by Ti and C based on the elemental distribution presented in Figure 9h,i. However, the content of Fe was less than that of Ti and C, which indicated that the diffusion of Fe was limited at lower temperature. Meanwhile, the fractural surface of Ti/steel was enriched with Fe (1.3–2.1 wt.%), Ti (79.2–80.8 wt.%) and C (19.1–19.8 wt.%) as indicated by WDS analysis. Thus, TiC may be formed on the fractural surface of Ti/steel based on the binary phase diagram of Ti and C.

The map distribution micrographs of various elements on the fractural surface of Ti/Nb/steel clad plate at heating temperature of 900 °C are exhibited in Figure 9. The BSE image of EPMA reveals that the fractural surface on the Ti side was almost covered with grey and white patterns (Figure 9a). Tearing ridge or dimple was not observed, which shows that the fractural mode of the clad interface was brittlement at heating temperature of 900 °C. In addition, Fe or C was not detected on the fractural surface of Ti side in Figure 9b,e which implied that the fractural position was not at the Nb/steel interface at heating temperature of 900 °C. However, the content of Ti was much higher than that of Fe and C on the fractural surface of Ti side in Figure 9c. Meanwhile, the content of Nb was also very high, especially in regions with low content of Ti (Figure 9d). It was considered that the fractural location of the clad interface was at the Ti/Nb interface, and was not between Nb/steel interface at heating temperature of 900 °C. The fractural surface of Ti side was enriched with Ti (58.4–59.1 wt.%), Nb (38.3–39.2 wt.%), Fe (0.1–0.8 wt.%), C (0.5–1.2 wt.%) and other trace elements based on WDS result. This noted that Nb-Ti solid solution may be formed at the clad interface instead of Ti-Fe compound. Thus, Nb-Ti solid solution had a significant influence on the performance of the clad plate at heating temperature of 900 °C.

**Figure 10:**
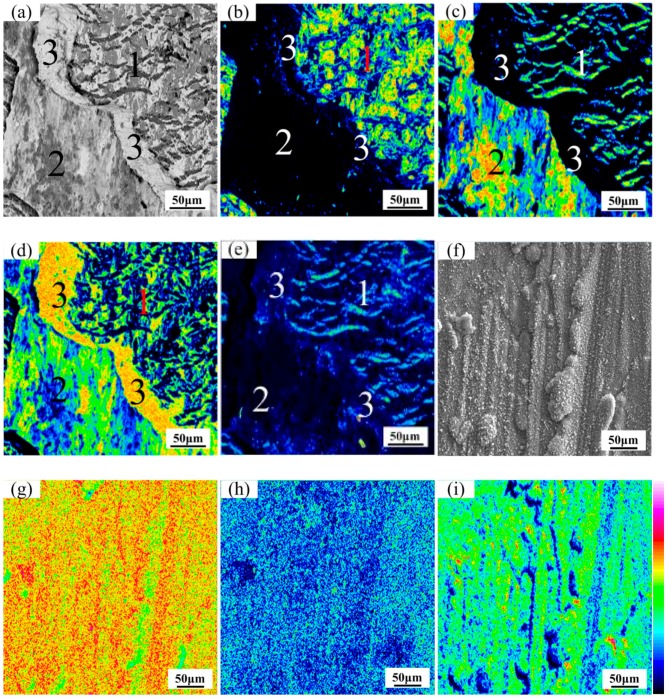
Map distribution micrographs of various elements on the fractural surface at 1000 °C (titanium side): (**a**) BSE image; (**b**) Fe element; (**c**) Ti element; (**d**) Nb element and (**e**) C element for Ti/Nb/steel clad plate; (**f**) BSE image; (**g**) Fe element; (**h**) Ti element and (**i**) C element for Ti/steel clad plate.

The map distribution micrographs of various elements on the fractural surface of Ti/Nb/steel clad plate at heating temperature of 1000 °C are presented in Figure 10a–e. The contents of Fe, Ti, C, and Nb were relatively high. The BSE image of the fractural surface on the Ti side can be divided into three regions in Figure 10a. The region 1 showed as-striped surface morphology, and the region 2 showed as-flat surface morphology with grey and black regions. However, the region 3 showed completely different surface morphology. The region 3 mainly contained Nb element (Figure 10d), which looks like a boundary that divides the elemental distribution micrograph into two parts. The region1 mainly contained Fe and Nb in Figure 10b,d, but Ti and C were distributed in the region with lower contents of Nb and Fe in Figure 10c,e. Thus, it can be speculated that Nb-Fe intermetallic compound and a small amount of TiC were formed in region 1. Meanwhile, the region 2 mainly contained Ti and Nb in Figure 10c,d, which implied that Nb-Ti solid solution may be formed on region 2. Thus, the fractural location of clad interface was at Nb-Ti solid solution (region 2), Nb interlayer (region 3), TiC and Nb-Fe intermetallic compound (region 1) at heating temperature of 1000 °C.

The map distribution micrographs of various elements on the fractural surface of Ti/steel clad plate at heating temperature of 1000 °C are illustrated in Figure 10f–i. The streak like tearing ridge was not found in BSE image of Figure 10f, and the fractural mode at the interface of Ti/steel clad plate was brittle. Meanwhile, the contents of Ti, Fe and C were high in Figure 10g–i. The fractural surface of Ti/steel was enriched with Ti (59.1–60.2 wt.%), Fe (20.1–20.9 wt.%), C (20.4–10.3 wt.%) and other trace elements according to WDS results. Thus, TiC, TiFe may be formed on the fractural surface of Ti/steel through the binary phase diagram of Ti, Fe and C, respectively.

**Figure 11:**
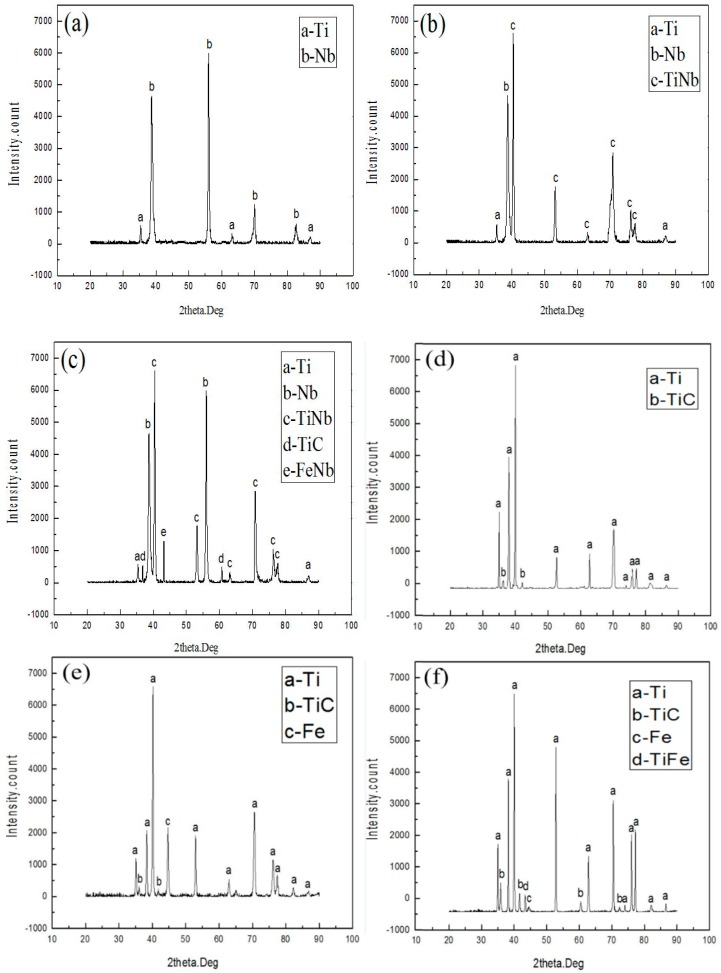
XRD results of the fractural surface at different heating temperatures (Ti side): (**a**) 800 °C; (**b**) 900 °C and (**c**) 1000 °C for Ti/Nb/steel clad plate; (**d**) 800 °C; (**e**) 900 °C and (**f**) 1000 °C for Ti/steel clad plate.

The XRD results of the fractural surface for Ti/Nb/steel clad plate at different heating temperatures are presented in Figure 11a–c. The fractural surface only contained Ti and Nb elements, and no intermetallic compound or solid solution was observed (Figure 11a). Thus, at heating temperature of 800 °C the shear fracture located between Ti and Nb interlayer or at the Nb interlayer, and is not between Nb/steel interface. The result was associated with the analysis of element distribution on the fractural surface at 800 °C. With increase of heating temperature, besides Ti and Nb phases, the Nb-Ti solid solution was also produced on the fractural surface of Ti side (Figure 11b). Thus, the fracture located between Ti and Nb-Ti solid solution, which corresponded with the analysis of element distribution on the fractural surface at 900 °C. However, TiC and Fe-Nb compounds were formed on the fractural surface of Ti side at 1000 °C. These results showed that the Nb interlayer cannot completely inhibit the diffusion of titanium elements, which caused the diffusion of Ti through Nb interlayer at heating temperature of 1000 °C. Some studies [28,29] identified that the sequence in the formation free energy of interfacial compounds was TiFe>TiFe_2_>β-Ti>TiC. Therefore, C element in steel and Ti diffused through Nb interlayer and then produced easily stable and discontinuous TiC. The previous studies [19,24] showed that discontinuous TiC and FeNb have a strong influence on the performance of clad plate. Therefore, the fractural surface of Ti side was divided into three parts, including the contact region between Nb and Ti, Nb interlayer and the contact region between Nb and steel in Figure 10c.

The XRD results of the fractural surface of Ti/steel clad plate at different heating temperatures are presented in Figure 11d–f. The fractural surface only contained α-Ti and TiC, and intermetallic compound was not formed on the fractural surface of Ti/steel clad plate in Figure 11d, which corresponded with the analysis of element distribution on the fractural surface at 800 °C. Meanwhile, α-Ti, TiC and α-Fe phases were formed at fractural surface at 900 °C in Figure 11e. With increase of heating temperature, Fe elements diffused to the interface and remained at the fractural surface of the Ti at 900 °C. However, the fractural surface not only contained α-Ti, α-Fe and TiC, but also had TiFe phase at 1000 °C in Figure 11f. It was indicated that Fe was further promoted and reacted with Ti to form TiC with increase of temperature. Under the combined action of both TiC and TiFe, the mechanical properties of Ti/steel clad plate were seriously influenced. Thus, Nb interlayer can inhibit the formation of TiC and TiFe at 800 and 900 °C, but Nb interlayer cannot inhibit formation of TiC at 1000 °C.

### 3.3. Shear Property of the Clad Interface

Table 3 reveals the shear properties of the Ti/Nb/steel interface at various heating temperatures. Increasing heating temperature led to the reduction in the interfacial shear strength. The sample clad at heating temperature of 800 °C exhibited maximum interfacial shear strength of ~279 MPa, while the minimum interfacial shear strength of ~152 MPa was achieved at heating temperature of 1000 °C. Thus, the drop in interfacial shear properties should be resulting from the occurrence of Nb-Ti solid solution, TiC and FeNb compounds. Table 4 represents the shear strength of the Ti/steel interface at various heating temperatures. The interfacial shear strength was decreased with increase of heating temperature. The maximum shear strength of ~249 MPa was produced in specimen clad at heating temperature of 800 °C, whereas the minimum value in interfacial shear strength of ~172 MPa was in the specimen at heating temperature of 1000 °C. Thus, shear strength of Ti/steel interface was seriously affected by joining action of TiC and TiFe.

**Figure 12:**
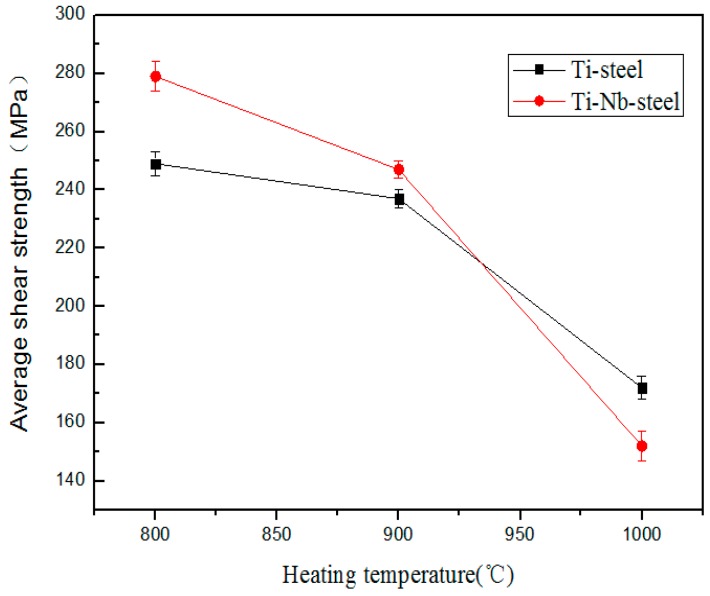
The shear properties of the Ti/Nb/steel and Ti/steel interfaces at different heating temperatures.

The shear properties of the Ti/Nb/steel and Ti/steel interfaces at different heating temperatures are shown in Figure 12. With increase of heating temperature, the difference in the interfacial shear strength was reduced. In addition, the shear strength of the Ti/steel interface was higher than that of the Ti/Nb/steel at 1000 °C, indicating that shear strength of Ti/Nb/steel interface was seriously influenced by TiC, FeNb and Nb-Ti solid solution. Obviously, shear strength in Ti/steel clad plate can be modified by introducing Nb interlayer at 800 and 900 °C.

**Figure 13:**
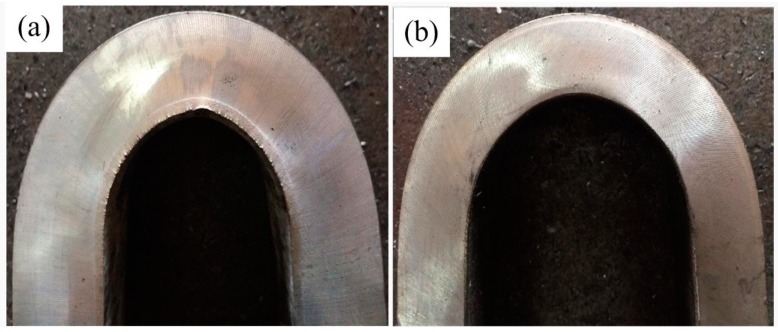
Bending test of the titanium clad steel plate with Nb interlayer: (**a**) Inner bending; (**b**) Outer bending.

The macrographs of clad plates after bending test are shown in the Figure 13. Ti/steel clad plates with Nb interlayer have excellent bending properties, and cracks are not formed at the interface after whether inner bending or outer bending.

When the vacuum brazing was subjected to cladding titanium and stainless steel adding Ag-28pct Cu alloy interlayer, the maximum value in interfacial shear strength reached to ~112 MPa at cladding temperature of 835 °C [30], while Ghosh et al. [14] reported that Ti/steel clad plate via diffusion bonding revealed an highest shear strength of 220 MPa. Furthermore, the shear strength in TA2/Q235B plate by hot-rolling bonding and inserting DT4 interlayer was up to 237.6 MPa [22]. However, the interfacial property in Ti/steel clad plate can be clearly increased by VRC and adding Nb interlayer.

The corrosion resistance of Ti/steel clad plate was not deteriorated by the addition of Nb interlayer because of good corrosion resistance of Nb. Thus, Nb interlayer can be used in the Ti/steel clad plates [16,21]. However, the price of Nb is relatively high. Therefore, during the industrial production of Ti/steel clad plate, it is very critical to minimize the amount of niobium interlayer on the basis of the Ti and steel isolation. It can be seen that the isolation effect of niobium interlayer is affected by the heating temperature and the thickness of the interlayer. Thus, in case of no effect on the mechanical property, it is useful to reduce the thickness of niobium at lower heating temperature.

## 4. Conclusions

The present work involved the determining role of Nb interlayer on the microstructural evolution and interfacial shear properties of Ti/steel plate by VRC at various heating temperatures. Some conclusions were obtained:

1. The inter-diffusion between Ti, C and Fe was effectively restrained by the Nb interlayer at heating temperature of 800 °C, and the Nb-Ti solid solution, TiC and Nb-Fe intermetallic compounds were not detected on the fractural surface. An average shear strength of 279 MPa was achieved.
2. At heating temperature of 900 °C, the inter-diffusion of Ti, C and Fe was remarkably restrained by the Nb interlayer, whereas Nb-Ti solid solution was formed at the Ti/Nb interface, which reduced the shear properties in the clad plate.
3. At heating temperature of 1000 °C, TiC and Nb-Fe compounds were formed at the Nb/steel interface, and the Nb-Ti solid solution was formed at the Ti/Nb interface. The mechanical properties of clad plates were seriously affected by those compounds. Minimum average shear strength of 152 MPa was gained.
4. The average shear strength of 249 and 172 MPa was obtained at Ti/steel interface at 800 and 1000 °C, respectively. The shear strength of the Ti/steel interface was higher than that of the Ti/Nb/steel interface at 1000 °C. Shear strength of Ti/steel clad plate can be modified by using VRC with Nb interlayer at 800 and 900 °C.

## Figures and Tables

**Table 1:** Chemical composition in wt.% of pure Ti and substrate steel.

Material	Ti	C	N	H	O	Fe	Mn	P	S	Si	Al	V
TA2	Bal	0.01	0.02	0.002	0.14	0.07	-	-	-	-	-	-
steel	-	0.2	-	-	-	Bal	1.2	0.025	0.015	0.55	0.02	0.05

**Table 2:** The detailed hot rolling and temperature parameters.

Pass	Pre-Rolling Thickness (mm)	Post-Rolling Thickness (mm)	Reduction (mm)	Reduction Ratio (%)	Temperature (°C)	Temperature (°C)	Temperature (°C)
1	60	45	15	25.00	800	900	1000
2	45	32	13	28.89	792	891	992
3	32	23	9	28.13	783	884	985
4	23	18	5	21.74	774	873	971
5	18	14	4	22.22	767	868	964
6	14	11	3	21.43	758	855	950
7	11	9	2	18.18	750	848	941

**Table 3:** The interfacial shear properties of Ti/Nb/steel clad plates at various heating temperatures.

Heating Temperature	Shear Strength, MPa						Average Shear Strength, MPa
800 °C	285	292	278	281	263	272	279
900 °C	127	234	251	245	257	270	247
1000 °C	151	174	147	145	168	125	152

**Table 4:** The interfacial shear properties of the Ti/steel clad plates at various heating temperatures.

Heating Temperature	Shear Strength, MPa						Average Shear Strength, MPa
800 °C	257	260	249	250	230	245	249
900 °C	216	225	246	230	242	265	237
1000 °C	169	196	169	163	191	142	172
